# Psychometric properties of the Internalized Stigma of Mental Illness (ISMI-10) scale in a Dutch sample of employees with mental illness

**DOI:** 10.1186/s12888-022-04284-5

**Published:** 2022-10-27

**Authors:** I. E. van Beukering, M. Bakker, R. I. Bogaers, K. M.E. Janssens, S. Gürbüz, M. C.W. Joosen, E. P.M. Brouwers

**Affiliations:** 1 Tranzo, Scientific Center for Care and Wellbeing, Tilburg School of Social and Behavioral Sciences, Tilburg University, P.O. box 90513, 5000 LE Tilburg, The Netherlands; 2 Netherlands Labour Authority, Den Haag, The Netherlands; 3 Department of Methodology and Statistics, Tilburg School of Social and Behavioral Sciences, Tilburg University, Tilburg, The Netherlands; 4 Brain Research and Innovation Centre, Ministry of Defense, Amsterdam, The Netherlands

**Keywords:** Internalized stigma, Mental illness, ISMI-10, Psychometric properties, Reliability, Validity, Dutch

## Abstract

**Background:**

Internalized stigma can have numerous negative effects on the well-being and employment of people with mental illness. Brief, valid, and reliable measures are needed to get a better understanding of self-stigmatization. The aim of this study is to translate the brief version of the Internalized Stigma of Mental Illness (ISMI-10) scale into a Dutch version and to assess the reliability and validity of this Dutch version in a sample of employees with mental illness.

**Methods:**

The ISMI-10 was translated into Dutch using the forward-backward translation procedure. The sample consisted of 161 employees with mental illness. Internal consistency was evaluated and the retest reliability was tested with 68 respondents. The construct validity was evaluated by testing convergent and divergent validity.

**Results:**

The Dutch ISMI-10 showed good internal consistency (α = 0.83) and good test-retest reliability (r = 0.73). The Dutch ISMI-10 demonstrated excellent convergent validity; high correlations were found between the Dutch ISMI-10 and hope (r = -0.54), anxiety and depression (r = 0.59), self-esteem (r = -0.56), and empowerment (r = − 0.59). Acceptable divergent validity was indicated; small correlations were found between the Dutch ISMI-10 and the physical functioning subscale (r = -0.27) and the role limitation due to physical problems subscale (r = -0.21), and medium correlations were found between the Dutch ISMI-10 and the general health subscale (r = -0.36).

**Conclusion:**

The Dutch ISMI-10 demonstrated adequate psychometric properties for assessing internalized stigma and can be used by researchers in Dutch speaking countries to get a better understanding of self-stigmatization among people with mental illness.

**Supplementary Information:**

The online version contains supplementary material available at 10.1186/s12888-022-04284-5.

## Background

Mental illness refers to a wide range of mental health conditions which can range in degree of severity (mild, moderate, or severe), such as depression, anxiety disorders, and schizophrenia [1]. Mental illness related stigma manifests itself in different ways. For instance, Corrigan and Bink (2005) refer to public stigma as “the process by which individuals in the general population first endorse the stereotypes of mental illness and then act in a discriminatory manner” [2]. Internalized stigma or self-stigma refers to a process of internalizing these expressed negative stereotypes by applying them to oneself which can lead to feelings of devaluation, lower self-esteem, marginalization, shame, withdrawal, and lower self-efficacy [3–5]. Mental illness related stigma can have numerous negative effects, like lower intentions to help-seeking, being more socially isolated, reduced psychiatric medication adherence, higher unemployment rates, and income loss [5–8]. More validated measures are urgently needed, especially in the employment setting where stigma has shown to be an important and underestimated contributing factor to unemployment [9, 10]. For example, both stigma by employers and internalized stigma have been found to be a problem for work participation [11–13], and the number of scientific publications on stigma and work has doubled in the past six years [10].

In the Netherlands, The Gatekeeper Improvement Act and the Extended Payment of Income Act are protecting disabled workers. The employer, the occupational physician, and the worker are responsible for the reintegration when a worker drops out due to sickness [14]. In the first two years of sickness absence the employers pay at least 70% of the income [15]. Employers are not allowed to ask about a health issue of a worker. The Netherlands has adopted a disability quota system which is called the Job Agreement. Government, municipalities, labor unions, and employers have agreed that they must supply 125,000 jobs for disabled persons by 2026. However, the labor participation rate among Dutch people with mental illness is low (21%) [16] and Dutch workers with mental illness are vulnerable to mental illness related stigma. For example, 64% of Dutch line managers are reluctant to hire workers with mental illness [11]. Also, a majority of Dutch workers expect lower chances of contract renewal and lower chances of getting a promotion after mental illness disclosure at work [17].

To get a better understanding of self-stigmatization there is a need for brief, valid, and reliable instruments that measures internalized stigma. The understanding and standardization of scales measuring internalized stigma is beneficial because it can lead to the development of effective (therapeutic) strategies to diminish internalized stigma. Worldwide, the most commonly used scale to measure internalized stigma is the Internalized Stigma of Mental Illness (ISMI) scale [18]. The ISMI is a self-report instrument which consists of 29 items and 5 subscales, with each item rated on a 1 (strongly disagree) to 4 (strongly agree) Likert scale. There are at least 55 known versions of the ISMI, these versions from all over the world showed reliability and validity across a diverse range of cultures and languages [19]. The ISMI has not yet been validated in the Netherlands. Because of the need for a shortened version to counter survey fatigue without losing quality, the developers of the ISMI created a brief version with 10 items, i.e. the brief version of the ISMI (ISMI-10) [20]. The ISMI-10 contains two items from every subscale of the original scale. The ISMI-10 questionnaire has already been translated, and the psychometric properties have been tested in countries like the Czech Republic, Greece, and Japan, however the questionnaire is not yet translated and tested in the Netherlands [21–23].

Therefore, the aim of this study is to translate the ISMI-10 into a Dutch version and to assess the reliability and validity of this Dutch version in a sample of employees with mental illness. Like in the original ISMI studies, a systematic review, and several validation studies, to evaluate convergent validity, it was hypothesized that the ISMI-10 will be negatively correlated with self-esteem and empowerment, and positively correlated with anxiety and depression [18–23]. It was also hypothesized that ISMI-10 will be negatively correlated with the concept of hope because internalized stigma is known to diminish self-esteem which has an adverse effect on hope [24]. To evaluate divergent validity, following the example of Van Gestel-Timmermans et al. (2010) [25], it was hypothesized no or only small correlations will be found with (physical) health.

## Methods

### Study design and participants

Data were collected using the Longitudinal Internet Studies for the Social Sciences (LISS) panel. This panel, administered by research institute CentERdata, consists of a representative and random selection of Dutch panel members who participate in monthly internet surveys covering different domains like work, education, housing, income, time use, political views, values, and personalities [26]. The LISS panel is based on a true probability sample of Dutch households drawn from the population register. All panel members gave written informed consent to participate in the surveys. More information about the LISS panel can be obtained at www.lissdata.nl.

The online questionnaire was sent in September 2021 to a selection of 322 panel members who indicated (1) to have mental illness (based on self-identification) in a previous LISS-survey, and (2) to have paid work. Having a mental illness was assessed by the question: “*Can you indicate whether you have a mental illness or not?*”, (1) yes or (2) no. For the present study, respondents were first asked whether they were still having mental illness, otherwise, they were excluded. The retest questionnaire, to assess the test-retest reliability, was sent five weeks after the data collection of the first questionnaire closed to a random selection of 75 respondents who had completed the first questionnaire (October/November 2021). Prior to its start, the Ethics Review Board of the School of Social and Behavioral Sciences of Tilburg University gave Ethical approval for this study (registration number: RP606). The Strengthening the Reporting of Observational Studies in Epidemiology guidelines were followed during the reporting of this study, all methods were carried out in accordance with these guidelines [27].

### Measures

#### Internalized stigma (Dutch ISMI-10)

##### ISMI-10

The ISMI-10 was developed to measure internalized stigma of mental illness. The ISMI-10 has 10 Likert scale items, with scores ranging from strongly disagree (1), disagree (2), agree (3), to strongly agree (4). Item 2 and item 9 are reversed scored. The original English version of the ISMI-10 showed adequate internal consistency (α = 0.75) [4]. There are three ways of interpreting the mean total scores. First, the score can be interpreted as a continuous variable, like in the original ISMI-10 paper [20]. Second, the 2-category method can be used to divide the scores into: does not report high internalized stigma (1.00-2.50) and reports high internalized stigma (2.51-4.00) [28]. Third, the 4-category method can be used to separate the scores into: minimal to no internalized stigma (1.00–2.00), mild internalized stigma (2.01–2.50), moderate internalized stigma (2.51-3.00), and severe internalized stigma (3.01-4.00) [29]. In this paper the score was interpreted the same as in the original ISMI-10 paper, as a continuous variable.

#### Translation and adaption of Dutch ISMI-10

The original English ISMI-10 was translated into Dutch following the forward-backward translation procedure [30]. First, the forward translation was performed by two subject matter experts (EB and MJ) with a good command of English and Dutch. They translated the English ISMI-10 scale into Dutch. This version was translated back into English by two native English speakers (who both also have a good command of Dutch). This backward translation was compared with the original English ISMI-10 based on conceptual balance. Any differences were discussed in detail between the subject matter experts (EB and MJ), native speakers, and authors (IB and MB) and necessary adjustments were made. Second, the preliminary validity was tested by conducting a pilot test with five (other) subject matter experts and five persons with mental illness to make sure that the Dutch version of the ISMI-10 was well understood in clarity and simplicity. The subject matter experts and the persons with mental illness were approached via the network of the researchers. During this procedure, only small additional changes were made to the initial translation, for example changing a word for a more relevant one. See Supplementary Material 1 for the final version of the Dutch ISMI-10.

#### Hope (HHI-Dutch)

The Herth Hope Index (HHI) is used for assessing the overall hope level of people with mental illness [31]. The HHI has 12 Likert scale items, ranging from strongly disagree (1) to strongly agree (4). Item 3 and item 6 were reversed-coded. The total scores range from 12 to 48, with higher scores indicating a higher level of hope. The original scale contains three factors: (1) temporality and future, (2) positive readiness and expectancy, and (3) interconnectedness with self and others. The overall reliability (α = 0.84) and validity of the Dutch version of the HHI (HHI-Dutch) was verified by Van Gestel-Timmermans et al. (2010) [25]. They advised to use the total scale scores instead of the subscales.

#### Anxiety and depression (HADS)

The Hospital Anxiety and Depression Scale (HADS) measures anxiety and depressive states [32]. The two subscales (anxiety and depression), each contain 7-items with Likert scores ranging from 0 to 3. The total scores of the two subscales range from 0 to 21, with higher scores indicating higher levels of anxiety and depression. The Dutch version of the HADS, used in this paper, has been validated for various groups of Dutch subjects and is stable across medical settings, and the internal consistency of the total scale for the different groups is good (α = 0.82–0.88) [33].

#### Self-esteem (Dutch RSES)

The Rosenberg Self-Esteem Scale (RSES) assesses global self-esteem [34]. This scale was also used in the original English ISMI-10 validation paper [20]. The RSES is designed as a unidimensional scale containing 10 Likert scale items ranging from strongly disagree (0) to strongly agree (3). Items 2, 5, 6, 8 and 9 were reversed scored. The total scores range from 0 to 30, with higher scores indicating a higher global self-esteem. The Dutch version of the RSES (Dutch RSES) proved to have good internal consistency (α = 0.86) and the validity was verified [35].

#### Empowerment (BUES-17)

The Boston University Empowerment Scale (BUES) measures empowerment [36]. In accordance with the original English ISMI-10 validation paper, the selection of the 17 items of factor 1 (self-esteem) and factor 2 (power) items were used (BUES-17), because the BUES-17 showed more support compared to the use of the whole scale (BUES-28) [20]. The BUES-17 contains items with scores ranging from strongly disagree (1) to strongly agree (4). Item 10 and item 17 were reverse coded. The total scores range from 0 to 64, with higher scores indicating more empowerment. Although not formally validated, the BUES-17 proved to have good internal consistency (α = 0.85) [20]. The BUES-17 was not formally tested for the Dutch context, there was one validation study that focused on the Dutch version of the original BUES with 28 items [37]. The Dutch version of the BUES-28 had a good internal consistency (α = 0.82). In the current study, the BUES-17 had a good internal consistency (α = 0.83).

#### Health (Dutch RAND-36)

The RAND-36 assesses health and contains eight subscales: physical functioning, social functioning, role limitations due to physical problems, role limitations due to emotional problems, emotional well-being, energy/fatigue, pain, and general health [38, 39]. The scale consists of items on 3-point to 6-point Likert scales and two subscales with 2-point Likert scales. Items 15, 17, and 19 were reversed-coded. The total scores range from 0 to 100, with a higher score indicating a more favorable health state. The internal consistency of the subscales and validity of the Dutch version of the RAND-36 (Dutch RAND-36) was verified by Van der Zee and Sanderman (2003) [40], and in accordance with Van Gestel-Timmermans et al. (2010) [8], the current study only used the 19 items of the subscales: physical functioning (α = 0.92), role limitations due to physical problems (α = 0.90), and general health (α = 0.81).

### Statistical analyses

Cronbach’s alpha was used to evaluate the reliability of the Dutch ISMI-10 by testing the internal consistency of the scale (α < 0.50 – unacceptable; α = 0.60–0.69 – questionable; α = 0.70–0.79 – acceptable; α = 0.80–0.89 – good; α > 0.90 – excellent) [41]. In addition, the internal consistency of the other scales used in this study was also evaluated. The test-retest reliability of the Dutch ISMI-10 was evaluated five weeks later after the data collection of the first questionnaire closed by assessing the scale with 75 respondents using Pearson’s correlation coefficients (r) between total scores (r = 0.40–0.59 – fair; r = 0.60–0.74 – good; r > = 0.75 – excellent) [42].

The construct validity was evaluated by computing the Pearson’s correlation coefficients (r) between the Dutch ISMI-10 and the HHI-Dutch, HADS, Dutch RSES, BUES-17, and Dutch RAND-36. Convergent validity was indicated by medium to high correlations with the HHI-Dutch, HADS, Dutch RSES, and BUES-17. Divergent validity was assumed if correlations were non-significant or small between the Dutch ISMI-10 and the RAND-36 subscales physical functioning, role limitations (due to physical problems), and general health. In line with Cohen [43], correlations of 0.10 to 0.29 were interpreted as small, 0.30 to 0.49 as medium and 0.50 to 1.0 as high.

Data of five respondents were excluded from analysis due to one or more missing values on the Dutch ISMI-10 items or the previous mentioned scales. All p-values below 0.05 were considered significant. All analyses were performed using SPSS Version 24.

## Results

### Sample characteristics

The online questionnaire was filled out by 256 of the 322 LISS-panel members who indicated (1) to have a mental illness in a previous LISS-survey, and (2) to have paid work (response rate = 79.5%). Of this group, 166 respondents belonged to the target group (employees who were still having mental illness) and 161 of them completed the questionnaire. The employees (60.2% female) had a mean age of 40.94 (SD = 12.14) years and had mostly a secondary (41.6%) or higher (45.3%) educational level. Other sample characteristics can be found in Table 1. The respondents mean internalized stigma score was 2.05 (SD = 0.44) and ranged from 1.00 to 3.20.

**Table 1:** Sociodemographic characteristics of the sample (N = 161)

	N (%)	M (SD)
Age in years		40.94 (12.14)
Gender		
Male	64 (39.8)	
Female	97 (60.2)	
Marital status		
Married	62 (38.5)	
Separated, divorced, or widowed	18 (11.2)	
Never married	81 (50.3)	
Domestic situation		
Single, with or without child(ren)	61 (37.9)	
(Un)married cohabitation, with or without child(ren)	96 (59.6)	
Other situation	4 (2.5)	
Educational level*		
Low	21 (13.0)	
Secondary	67 (41.6)	
High	73 (45.3)	
Gross household income per month (in euro)**		4480 (2520)

*Based on the highest level of education completed. ‘Low’ (primary school, intermediate secondary, US: junior high school), ‘secondary’ (higher secondary education/preparatory university education, US; senior high school; intermediate vocational education, US; junior college), and ‘high’ (higher education, US: college; university)

** Information not available for all respondents (11 missings)

### Reliability analysis

#### Internal consistency

The Dutch ISMI-10 had a good internal reliability of α = 0.83. Table 2 shows the Cronbach’s alpha of the overall scale of the Dutch ISMI-10 compared to the overall scales of the short original English version (ISMI-10) and the original English version (ISMI-29) [18, 20]. In this present study, this were the Cronbach’s alpha coefficients for the other used scales: HHI-Dutch (α = 0.81), HADS (α = 0.90), Dutch RSES (α = 0.89), BUES-17 (α = 0.83) and RAND-36 subscales (physical functioning: α = 0.92; role limitations – physical problems: α = 0.83; general health: α = 0.80).

**Table 2:** Cronbach’s alpha coefficients in relation to the internal consistency of the Dutch ISMI-10, original English ISMI-10, and original English ISMI-29

	Dutch ISMI-10	Original English ISMI-10	Original English ISMI-29
Overall scale	0.83	0.75	0.90

#### Test-retest reliability

The test-retest study involved 63 of 75 respondents (response rate = 84.0%) who completed the questionnaire after 37 to 82 days (average 56.2 days). No significant differences were found on sociodemographic characteristics between the retest sample compared to the total sample. The test-retest study showed good test-retest reliability of r = 0.73 (p < 0.01).

### Construct validity

#### Convergent validity

Convergent validity was assessed by computing the correlating between the Dutch ISMI-10 sum scores and the HHI-Dutch, HADS, Dutch RSES, and BUES-17. Table 3 shows the correlations. High significant correlations were found between the Dutch ISMI-10 and HHI-Dutch, HADS, Dutch RSES, and BUES-17 (p < 0.01).

**Table 3:** Pearson correlations of the Dutch ISMI-10 with HHI-Dutch, HADS, Dutch RSES, and BUES-17 to assess convergent validity

	N	HHI-Dutch	HADS	Dutch RSES	BUES-17
Dutch ISMI-10	161	-0.54**	0.59**	-0.56**	-0.58**

** P < 0.01 *P < 0.05

#### Divergent validity

Divergent validity was evaluated by correlating Dutch ISMI-10 sum scores with the RAND-36 subscales physical functioning, role limitations due to physical problems, and general health. Table 4 shows that small significant correlations were found between the Dutch ISMI-10 and the physical functioning subscale and the role limitation subscale (p < 0.01). Medium significant correlations were found between the Dutch ISMI-10 and the general health subscale (p < 0.01).

**Table 4:** Pearson correlations of the Dutch ISMI-10 with three RAND-36 subscales to assess divergent validity

	N	RAND- 36 Physical functioning subscale	RAND- 36 Role limitations due to physical problems subscale	RAND-36 General health subscale
Dutch ISMI-10	161	-0.27**	-0.21**	-0.36**

** P < 0.01 *P < 0.05

## Discussion

The aim of this current study was to translate the ISMI-10 into a Dutch version and to assess the reliability and validity in a sample of employees with mental illness. A Dutch version of the ISMI-10 was translated using the forward-backward translation procedure [30]. The Dutch ISMI-10 showed good internal consistency and good test-retest reliability. The Dutch ISMI-10 showed excellent convergent validity and acceptable divergent validity.

Analysis of the reliability of the Dutch ISMI-10 supported the reliability of the questionnaire. The internal reliability of the Dutch ISMI-10 was better compared to the original English ISMI-10, but slightly lower than the original English ISMI-29. One explanation for this difference is that the alpha is mostly higher in scales with more items [44]. This was also seen in the Czech and original English ISMI-10 papers [20, 21]. The Dutch ISMI-10 showed similar test-retest reliability compared to the results from the Japanese ISMI-10, which is the only version that studied test-retest reliability [23].

As expected, we found high negative correlations between the Dutch ISMI-10 and hope (HHI-Dutch), self-esteem (Dutch RSES), and empowerment (BUES-17), and positive correlations with anxiety and depression (HADS). These convergent validity findings are in line with the findings of the papers concerning the original English ISMI-29, the original English ISMI-10, and the Japanese version of the ISMI-10 using self-esteem, empowerment, and depression measures [18, 20, 23]. Additional to other ISMI-10 papers, this current study also used an extra measure, a measure for hope, to test convergent validity thoroughly.

Analysis of the divergent validity showed that, as expected, small correlations were found between the Dutch ISMI-10 and the RAND-36 subscales physical functioning and role limitations due to physical problems, but unexpected medium correlations were found with the general health subscale. The medium correlation with the general health subscale may be explained by the fact that general health consists of both a physical and a mental element. Especially in a sample of respondents with mental illness, this mental element might be experienced as more prevailing [25]. For future psychometric research on the ISMI-10 and divergent validity, this subscale may be less suitable in respondents with mental illness.

In contrast to several ISMI-29 studies [18, 45, 46], this study did not include a factor analysis to investigate the factor structure of the scale, because the ISMI-10 is meant to be an unidimensional scale [20]. Although the ISMI-10 includes all of the five dimensions in two-item subscales, the developers of the original English ISMI-10 did not intend to make five subscales. The developer mentioned that two-item scales are likely to be unstable [20]. Other research showed that for multidimensional scales, scales need at least four items to use Confirmative Factor Analysis for validation [47]. A later study on the factor structure of different versions of the ISMI confirmed the recommendation of the developers and underlined the one-dimensionality of the ISMI-10 [48]. Thus, we interpreted the ISMI-10 as a unidimensional scale.

### Strengths and limitations

A strength of this study, compared to the original ISMI-10 paper, was the test-retest design to describe the stability of the Dutch ISMI-10. Another strength was the study sample, which consisted of a selection of employees with mental illness from a representative panel of the Dutch population. Some of the psychometric validation studies of the ISMI have used more specific samples, such as the original ISMI-10, which included a male military veterans sample who received Veterans Affairs services [20]. However, the current sample did not include unemployed people with mental illness, which may be a limitation because this group has been found to have higher levels of self-stigma compared to their employed counterparts [49]. Other ISMI-10 validation papers from Greece, Japan, and Czech Republic focused on mental illness outpatients in general [21–23]. The overall Dutch ISMI-10 mean score (2.05, SD = 0.44) is indeed slightly lower than the reported overall ISMI-10 mean scores of the original ISMI-10 paper (2.32, SD = 0.39) and the Czech ISMI-10 paper (2.16, SD = 0.54) [20, 21]. Another limitation of this study was the use of self-report data to determine if the respondent experienced mental illness at the time of the data collection, and the use of data that did not include more background information on participants’ diagnoses, symptom severity or participants’ working situations. An additional limitation was the use of the BUES-17 scale for assessing convergent validity. This shorter version of the BUES-28 scale was not formally tested for the Dutch context. Nevertheless, the translated Dutch version of the BUES-17 scale proved to have good internal consistency in this study. Additionally, the HADS-scale is originally developed for medical out-patients, in this study the HADS-scale was used for workers following the example of other studies [50–52]. To conclude, a limitation could be the use of simple correlations to check convergent and divergent validity. A more rigorous analytical procedure could have been used, e.g. examining latent construct correlations [53].

## Conclusion

In conclusion, the results of this study revealed that the Dutch ISMI-10 demonstrated adequate psychometric properties. The Dutch ISMI-10 showed good internal consistency, good test-rest reliability, excellent convergent validity, and acceptable divergent validity. The ISMI-10 had already been shown to be a good and brief alternative for the original ISMI-29 [20]. The understanding and standardization of scales measuring internalized stigma like the Dutch ISMI-10 can be used by researchers in Dutch speaking countries to get a better understanding of self-stigmatization among people with mental illness. The Dutch ISMI-10 is valuable for the development of effective (therapeutic) strategies to diminish internalized stigma.

## Electronic supplementary material

Below is the link to the electronic supplementary material.

Supplementary Material 1. Dutch ISMI-10

## Data Availability

The datasets generated and analysed during the current study are not publicly available, due to the use of the data in other research papers which are not published yet but are available from the corresponding author on reasonable request.

## References

[1] National Institute of Mental Health [Internet]. 2022 [Definitions]. Available from: https://www.nimh.nih.gov/health/statistics/mental-illness#part_2538.

[2] Corrigan P, Bink AB. On the stigma of mental illness. American Psychological Association.; 2005.

[3] Corrigan PW, Watson AC (2002). The paradox of self-stigma and mental illness. Clin Psychol Sci Pract.

[4] Corrigan PW (1998). The impact of stigma on severe mental illness. Cogn Behav Pract.

[5] Livingston JD, Boyd JE (2010). Correlates and consequences of internalized stigma for people living with mental illness: A systematic review and meta-analysis. Soc Sci Med.

[6] Corrigan P (2004). How stigma interferes with mental health care. Am Psychol.

[7] Yanos PT, Roe D, Markus K, Lysaker PH (2008). Pathways between internalized stigma and outcomes related to recovery in schizophrenia spectrum disorders. Psychiatric Serv.

[8] Link B. Mental patient status, work, and income: An examination of the effects of a psychiatric label. American Sociological Review. 1982:202–15. 7091929

[9] Brouwers EPM. Social stigma is an underestimated contributing factor to unemployment in people with mental illness or mental health issues: position paper and future directions. 2020;8:1–7. 10.1186/s40359-020-00399-0 PMC7171845 32317023

[10] Van Beukering I, Smits S, Janssens K, Bogaers R, Joosen M, Bakker M, et al. In what ways does health related stigma affect sustainable employment and well-being at work? A systematic review. Journal of occupational rehabilitation. 2021:1–15. 10.1007/s10926-021-09998-z PMC9576674 34487290

[11] Janssens KM, van Weeghel J, Dewa C, Henderson C, Mathijssen JJ, Joosen MC, et al. Line managers’ hiring intentions regarding people with mental health problems: a cross-sectional study on workplace stigma. Occupational and Environmental Medicine; 2021. 10.1136/oemed-2020-106955 PMC8292579 33542095

[12] Corrigan PW, Larson JE, Ruesch NJWp. Self-stigma and the “why try” effect: impact on life goals and evidence-based practices. 2009;8(2):75. 10.1002/j.2051-5545.2009.tb00218.x PMC2694098 19516923

[13] Corrigan PW, Powell KJ, Rüsch N. How does stigma affect work in people with serious mental illnesses? 2012;35(5):381. 10.1037/h0094497 23116379

[14] de Rijk A. Work disability prevention in the Netherlands: a key role for employers. The science and politics of work disability prevention. Routledge; 2018. pp. 223–41.

[15] Arends I (2014). Mental Health.

[16] Boumans J, Michon H, Hulsbosch L, Knispel A, de Lange A. Achterblijvende arbeidsparticipatie onder mensen met psychische problemen. Perspectieven van panelleden en re-integratieprofessionals Deelonderzoek. 2018.

[17] van Beukering I, Bakker M, Corrigan P, Gürbüz S, Bogaers R, Janssens K, et al. Expectations of Mental Illness Disclosure Outcomes in the Work Context: A Cross-Sectional Study Among Dutch Workers. Journal of Occupational Rehabilitation. 2022:1–12. 10.1007/s10926-022-10026-x PMC9668951 35137273

[18] Ritsher JB, Otilingam PG, Grajales M (2003). Internalized stigma of mental illness: psychometric properties of a new measure. Psychiatry Res.

[19] Boyd JE, Adler EP, Otilingam PG, Peters T (2014). Internalized Stigma of Mental Illness (ISMI) scale: a multinational review. Compr Psychiatr.

[20] Boyd JE, Otilingam PG, DeForge BR (2014). Brief version of the Internalized Stigma of Mental Illness (ISMI) scale: psychometric properties and relationship to depression, self esteem, recovery orientation, empowerment, and perceived devaluation and discrimination. Psychiatr Rehabil J.

[21] Ociskova M, Prasko J, Kamaradova D, Marackova M, Holubova M (2016). Evaluation of the psychometric properties of the brief Internalized Stigma of Mental Illness Scale (ISMI-10). Neuroendocrinol Lett.

[22] Paraskevoulakou A, Vrettou K, Pikouli K, Triantafillou E, Lykou A, Economou M (2017). Mental illness related internalized stigma: Psychometric Properties of the Brief ISMI Scale in Greece. Materia socio-medica.

[23] Tanabe Y (2021). Validation of the 10-item Internalized Stigma of Mental Illness Scale: Validation of the Japanese Version. J Japan Acad Psychiatric Mental Health Nurs.

[24] Corrigan PW, Rafacz J, Rüsch N (2011). Examining a progressive model of self-stigma and its impact on people with serious mental illness. Psychiatry Res.

[25] Van Gestel-Timmermans H, Van Den Bogaard J, Brouwers E, Herth K, Van Nieuwenhuizen C (2010). Hope as a determinant of mental health recovery: a psychometric evaluation of the Herth Hope Index‐Dutch version. Scand J Caring Sci.

[26] Scherpenzeel AC (2018). “True” Longitudinal and Probability-Based Internet Panels: Evidence From the Netherlands.

[27] Von Elm E, Altman DG, Egger M, Pocock SJ, Gøtzsche PC, Vandenbroucke JP (2007). The Strengthening the Reporting of Observational Studies in Epidemiology (STROBE) statement: guidelines for reporting observational studies. Bull World Health Organ.

[28] Ritsher JB, Phelan JC (2004). Internalized stigma predicts erosion of morale among psychiatric outpatients. Psychiatry Res.

[29] Lysaker PH, Roe D, Yanos PT (2007). Toward understanding the insight paradox: internalized stigma moderates the association between insight and social functioning, hope, and self-esteem among people with schizophrenia spectrum disorders. Schizophr Bull.

[30] Brislin RW. The wording and translation of research instruments. 1986.

[31] Herth K (1992). Abbreviated instrument to measure hope: development and psychometric evaluation. J Adv Nurs.

[32] Zigmond AS, Snaith RP (1983). The hospital anxiety and depression scale. Acta psychiatrica Scandinavica.

[33] Spinhoven P, Ormel J, Sloekers P, Kempen G, Speckens AE, van Hemert AM (1997). A validation study of the Hospital Anxiety and Depression Scale (HADS) in different groups of Dutch subjects. Psychol Med.

[34] Rosenberg M (1979). Conceiving the Self.

[35] Franck E, De Raedt R, Barbez C, Rosseel Y (2008). Psychometric properties of the Dutch Rosenberg self-esteem scale. Physiol Belgica.

[36] Rogers ES, Chamberlin J, Ellison ML (1997). Measure empowerment among users of mental health services. Psychiatric Serv.

[37] Boevink W, Kroon H, Delespaul P, Van Os J (2016). Empowerment according to persons with severe mental illness: development of the Netherlands empowerment list and its psychometric properties. Open J Psychiatry.

[38] Ware JE Jr, Sherbourne CD. The MOS 36-item short-form health survey (SF-36): I. Conceptual framework and item selection. Medical care. 1992:473 – 83. 1593914

[39] Hays RD, Sherbourne CD, Mazel RM (1993). The rand 36-item health survey 1.0. Health Econ.

[40] Van der Zee K, Sanderman R. Het meten van de algemene gezondheidstoestand met de RAND-36: een handleiding. Groningen: Noordelijk centrum voor gezondheidsvraagstukken. 1993:1–23.

[41] George DM, Mallery EP (2003). SPSS for Windows step by step: A simple guide and reference. 11.0 update.

[42] Cicchetti DV (1994). Guidelines, criteria, and rules of thumb for evaluating normed and standardized assessment instruments in psychology. Psychol Assess.

[43] Cohen J. Statistical power analysis for the behavioral sciences. Academic press; 2013.

[44] Cortina JM (1993). What is coefficient alpha? An examination of theory and applications. J Appl Psychol.

[45] Tanabe Y, Hayashi K, Ideno Y (2016). The Internalized Stigma of Mental Illness (ISMI) scale: validation of the Japanese version. BMC Psychiatry.

[46] Hwang TY, Lee WK, Han ES, Kwon EJ (2006). A study on the reliability and validity of the Korean version of internalized stigma of mental illness scale (K-ISMI). J Korean Neuropsychiatric Association.

[47] Knekta E, Runyon C, Eddy S (2019). One size doesn’t fit all: Using factor analysis to gather validity evidence when using surveys in your research. CBE—Life Sci Educ.

[48] Hammer JH, Toland MD (2017). Internal structure and reliability of the Internalized Stigma of Mental Illness Scale (ISMI-29) and Brief Versions (ISMI-10, ISMI-9) among Americans with depression. Stigma and Health.

[49] Brohan E, Elgie R, Sartorius N, Thornicroft G, Group G-ES (2010). Self-stigma, empowerment and perceived discrimination among people with schizophrenia in 14 European countries: The GAMIAN-Europe study. Schizophr Res.

[50] Heesakkers H, Zegers M, van Mol MM, van den Boogaard M (2021). The impact of the first COVID-19 surge on the mental well-being of ICU nurses: A nationwide survey study. Intensive and Critical Care Nursing.

[51] Hamano J, Tachikawa H, Takahashi S, Ekoyama S, Nagaoka H, Ozone S (2022). Exploration of the impact of the COVID-19 pandemic on the mental health of home health care workers in Japan: a multicenter cross-sectional web-based survey. BMC Prim Care.

[52] d’Ussel M, Fels A, Durand X, Lemogne C, Chatellier G, Castreau N (2022). Factors associated with psychological symptoms in hospital workers of a French hospital during the COVID-19 pandemic: Lessons from the first wave. PLoS ONE.

[53] Hodson G. Construct jangle or construct mangle? Thinking straight about (nonredundant) psychological constructs. Journal of Theoretical Social Psychology. 2021.

