# Supplementary material for: Psychometric properties of the Internalized Stigma of Mental Illness (ISMI-10) scale in a Dutch sample of employees with mental illness
Source: BMC Psychiatry. 2022 Oct 27;22:662. doi: 10.1186/s12888-022-04284-5 (PMC9609266; doi:10.1186/s12888-022-04284-5)
Supplement: Supplementary file 1 — Supplementary Material 1. Dutch ISMI-10 [file 12888_2022_4284_MOESM1_ESM.docx]

**Supplementary Material 1: Dutch ISMI-10**

This is the Dutch version of the ISMI-10 along with the original English statements.

| Dutch ISMI-10 | | | | |
| --- | --- | --- | --- | --- |
| Geef voor elke stelling aan of u het er (1) sterk mee oneens, (2) oneens, (3) eens of (4) sterk mee eens bent. | Sterk oneens  (*Strongly disagree*) | Oneens  (*Disagree*) | Eens  (*Agree*) | Sterk mee eens  (*Strongly agree*) |
| 1. Mensen met psychische aandoeningen hebben de neiging gewelddadig te zijn   (*Mentally ill people tend to be violent*) | 1 | 2 | 3 | 4 |
| 1. Mensen met psychische aandoeningen leveren een belangrijke bijdrage aan de maatschappij   (*People with mental illness make important contributions to society*) | 1 | 2 | 3 | 4 |
| 1. Ik ga minder om met anderen als ik gewend was omdat ik door mijn psychische aandoening er mogelijk “raar” uitzie of mij “raar” gedraag   (*I don’t socialize as much as I used to because my mental illness might make me look or behave “weird*) | 1 | 2 | 3 | 4 |
| 1. Het hebben van een psychische aandoening heeft mijn leven verpest   (*Having a mental illness has spoiled my life*) | 1 | 2 | 3 | 4 |
| 1. Ik ga sociale situaties uit de weg om mijn familie en vrienden niet in verlegenheid te brengen   (*I stay away from social situations in order to protect my family or friends from embarrassment*) | 1 | 2 | 3 | 4 |
| 1. Mensen zonder psychische aandoeningen kunnen mij onmogelijk begrijpen   (*People without mental illness could not possibly understand me*) | 1 | 2 | 3 | 4 |
| 1. Mensen negeren mij of nemen mij minder serieus, enkel omdat ik een psychische aandoening heb   (*People ignore me or take me less seriously just because I have a mental illness*) | 1 | 2 | 3 | 4 |
| 1. Ik kan niets bijdragen aan de maatschappij omdat ik een psychische aandoening heb   (*I can’t contribute anything to society because I have a mental illness*) | 1 | 2 | 3 | 4 |
| 1. Ik kan een goed, volwaardig leven hebben, ondanks mijn psychische aandoening   (*I can have a good, fulfilling life, despite my mental illness*) | 1 | 2 | 3 | 4 |
| 1. Anderen denken dat ik niet veel in het leven kan bereiken omdat ik een psychische aandoening heb   (*Others think that I can’t achieve much in life because I have a mental illness*) | 1 | 2 | 3 | 4 |
